# Reply to Comment on Małgorzata Krówczyńska and Ewa Wilk. Environmental and Occupational Exposure to Asbestos as a Result of Consumption and Use in Poland. *Int. J. Environ. Res. Public Health* 2019, 16, 2611

**DOI:** 10.3390/ijerph17062145

**Published:** 2020-03-24

**Authors:** Małgorzata Krówczyńska, Ewa Wilk

**Affiliations:** Department of Geoinformatics, Cartography and Remote Sensing, Chair of Geomatics and Information Systems, Faculty of Geography and Regional Studies, University of Warsaw, 00-927 Warsaw, Poland; ewa.wilk@uw.edu.pl

Thank you for the possibility of enabling us to provide a response to the comment on our paper “Environmental and Occupational Exposure to Asbestos as a Result of Consumption and Use in Poland” [1].

The National Cancer Registry [2] cited in our publication was created as a response to the implementation of the Polish Act on the information system in healthcare (Journal of Laws No. 113 of 2011, item 657). One of the crucial modules implemented in the National Cancer Registry was the creation of a central, nationwide database of cancer cases, to which employees of provincial medical centers were provided with online access through a dedicated application. This led to the elimination of double recording of patients in the database, elimination of differences in patient information resulting from data dispersion, and provided access to common dictionary databases. A system of communication between registers was created. The security element is the database access authorization system. Meeting the requirements of the Act on the information system in healthcare is enabling data to be obtained electronically from all external data sources. Opportunities for keeping organ registers were also created to assess the effectiveness of cancer treatment in Poland. That is why this source of information was assessed as reliable and data was gathered to present the actual state of malignant mesothelioma cases; moreover they are distinguished in the aforementioned database. Malignant mesothelioma is classified according to the International Statistical Classification of Diseases and Related Health Problems ICD-10 as C.45 [3] and constituted the subject of the undertaken survey as a unique indicator of asbestos exposure [4]. The National Cancer Registry enables the gathering of data on a local level, which is time consuming; nevertheless, it supports the possibility of analyzing data on a local level with reference to the type of cancer and administrative units. In our opinion, all necessary data were used in order to provide reliable information since the National Cancer Registry is conducted on a state level, i.e., it administered by the Ministry of Health fulfilling legal obligations. That is why we may not agree with the statement put in the comment *“The only available, reliable source on the origin of mesothelioma is occupational exposure data”.* This is a too-far-reaching conclusion which is not supported by the evidence and the use of a national register that is kept in accordance with the applicable law for the whole country is a highly desirable action, which was actually presented in our paper.

Data on the absolute number of malignant mesothelioma cases were derived from the Report for the Ministry of Economy [5], which constituted the basis for the analysis of asbestos exposure and the mesothelioma incidence in Poland [6]. This report was prepared on the basis of data provided by the Nofer Institute of Occupational Medicine for the Polish Ministry of Economy, who financed the aforementioned survey. Therefore, data gathered during the project conducted by the Nofer Institute of Occupational Medicine for the Ministry of Economy and the Ministry of Health should be considered reliable. The number of malignant mesothelioma cases analyzed in the aforementioned report and, as a result, in our paper, was also provided and confirmed by Szeszenia-Dąbrowska et al. (from the Nofer Institute of Occupational Medicine, including Beata Świątkowska) [7] and amounted to 138, as analyzed in our paper. The same explanation is provided for the number of medical examinations, which is raised in the comment. Nevertheless, there are discrepancies between the data presented by the Nofer Institute of Occupational Medicine in various publications, including reports presented to the Ministry of Health and the Ministry of Economy [5,6], who financed those experiments. We hope that the Nofer Institute of Occupational Medicine will work on making the data available online, which will make it reliable, unambiguous, and possible for analysis for a wider group of researchers.
